# Whole Genome Sequence of an Edible Mushroom *Oudemansiella raphanipes* (Changgengu)

**DOI:** 10.3390/jof9020266

**Published:** 2023-02-16

**Authors:** Liping Zhu, Xia Gao, Meihua Zhang, Chunhui Hu, Wujie Yang, Lizhong Guo, Song Yang, Hailong Yu, Hao Yu

**Affiliations:** 1 Shandong Provincial Key Laboratory of Applied Mycology, School of Life Sciences, Qingdao Agricultural University, 700 Changcheng Road, Chengyang District, Qingdao 266109, China; 2 Institute of Edible Fungi, Shanghai Academy of Agricultural Sciences, National Engineering Research Center of Edible Fungi, Shanghai 201403, China; 3 Shandong Agricultural Technology Extending Station, Jinan 250100, China

**Keywords:** *Oudemansiella raphanipes*, genome, monokaryon, secondary metabolites, CAZyme, phylogenetic analysis

## Abstract

*Oudemansiella raphanipes*, considered as a well-known culinary edible mushroom with a high content of natural bioactive substances, is widely cultivated in China with the commercial name Changgengu. However, due to the lack of genomic data, molecular and genetic study on *O. raphanipes* is rare. To obtain a comprehensive overview of genetic characteristics and enhance the value of *O. raphanipes*, two mating-compatible monokaryons isolated from the dikaryon were applied for de novo genome sequencing and assembly using Nanopore and /or Illumina sequencing platforms. One of the monokaryons, *O. raphanipes* CGG-A-s1, was annotated with 21,308 protein-coding genes, of which 56 were predicted to be involved in the biosynthesis of secondary metabolites such as terpene, type I PKS, NRPS, and siderophore. Phylogenetic and comparative analysis of multiple fungi genomes revealed a close evolutionary relationship between *O. raphanipes* and *Mucidula mucid* based on single-copy orthologous protein genes. Significant collinearity was detected between *O. raphanipes* and *Flammulina velutipes* on the synteny of inter-species genomes. 664 CAZyme genes in CGG-A-s1 were identified with GHs and AAs families significantly elevated when compared with the other 25 sequenced fungi, indicating a strong wood degradation ability. Furthermore, the mating type locus analysis revealed that CGG-A-s1 and CGG-A-s2 were conserved in the gene organization of the mating *A* locus but various in that of the mating *B* locus. The genome resource of *O. raphanipes* will provide new insights into its development of genetic studies and commercial production of high-quality varieties.

## 1. Introduction

*Oudemansiella raphanipes* is a well-known culinary edible mushroom with an excellent unique flavor, as well as a medicinal mushroom with high economic value [1,2]. In China, it was originally produced in Yunnan province and was commercially known as Changgengu or Heipijizong [2]. As for the taxonomic name, it was first described by M. J. Berkeley in India and effectively published in 1850 named as *Agaricus raphanipes* [2]. However, it was confusingly classified as *Hymenopellis* raphanipes, *Hymenopellis* furfuracea, *Oudemansiella furfuracea,* or *Oudemansiella radicata* in China during the past decades [1,2,3], but until 2016 its classification was determined to be *O. raphanipes* based on morphological and molecular data [2]. Furthermore, no *O. radicata* or *O. furfuracea* species were detected within over 300 samples collected from 13 provinces in China since 2013 [2], indicating that these two previously reported species should also classified as *O. raphanipes*. Since then, *O. raphanipes* has been used to refer to Heipijizong in domestic academic research to avoid naming confusion.

*O. raphanipes* has been recently cultivated throughout China since its artificial cultivation in large quantities was realized [3]. Numerous bioactive compounds produced by *O. raphanipes* have been discovered, including polysaccharides, enzymes, orcinol, ergosterol, triterpenes, and other nutrients, indicating that it plays a positive role in antioxidant, antitumor, immunomodulatory, and hepatoprotection [4,5,6,7]. Owing to its outstanding health-promoting properties, multiple studies have been focused on domestication and cultivation conditions, the liquid fermentation process, bioactive compound extraction, and medical function identification in *O. raphanipes* [6,7,8,9,10,11]. The huge demand makes *O. raphanipes* commercial cultivation profitable. However, molecular biological and genetic study on *O. raphanipes* is rare due to the lack of genomic information, which limits its development of molecular genetic features.

As sequencing technology advances, genome sequencing has been extensively applied to investigate the genetic traits of mushrooms [12,13,14]. In particular, numerous genomes of mushrooms, including *Pleurotus giganteus* [15], *Clitopilus passeckerianus* [16], *Stropharia rugosoannulata* [17], *Gomphus purpuraceus* [18], *Inonotus obliquus* [19], *Inonotus hispidus* [20], and *Laetiporus sulphureus* [21], among others, have been most recently reported, which will provide valuable genetic resources and molecular markers for biological and genetic studies of edible mushrooms and also disease. Therefore, the availability of high-quality whole genomic sequencing for *O. raphanipes* is imperative to carry out, as it would facilitate the identification of functional candidate genes and genetic breeding of the cultivars.

In the present study, the complete genomes of *O. raphanipes* CGG-A-s1 and *O. raphanipes* CGG-A-s2 were sequenced using Illumina and Nanopore sequencing platforms. Comparative analysis was conducted between the genome of *O. raphanipes* CGG-A-s1 and the other 25 published fungi genomes. Secondary metabolite-related genes, carbohydrate-active enzymes (CAZymes), and mating genes were also analyzed. The availability of a whole-genome sequence will help to classify the taxonomic status of *O. raphanipes* and aid future breeding efforts to improve its commercial cultivation.

## 2. Materials and Methods

### 2.1. Strains and Culture Conditions

The *O. raphanipes* strain CGG-A was provided by the Laboratory of Mushroom Precision Breeding (http://mushroomlab.cn/, accessed on 1 December 2022). The *O. raphanipes* strain was cultivated and maintained on potato dextrose agar (PDA) plates. For fruiting body production, the strain was inoculated into solid media (60% (*w/w*) sawdust, 20% (*w/w*) corncob, 18% (*w/w*) wheat bran, 1% (*w/w*) sucrose and 1% (*w/w*) gypsum powder) in a polypropylene bag. Vegetative growth of *O. raphanipes* mycelia was carried out at 25 °C with a humidity of 70%. After 35 to 40 days of cultivation, the mycelia occupied the full culture bag. The mycelia then continued cultivation under the same conditions for 30 to 35 days for maturation. The polypropylene bag was removed and the solid culture was buried into the ground and covered by 3–4 cm soil. At 20 to 30 days after earthing, the fruiting body formed under the stimulation of temperature, water, and light. The basidiospores were collected and the monokaryotic strains CGG-A-s1 and CGG-A-s2 were isolated from the spores as previously described [15].

### 2.2. Genome and Transcriptome Sequencing

The *O. raphanipes* CGG-A-s1 and CGG-A-s2 strains were inoculated on PDA plates covered with cellophane. The mycelia were scraped from the cellophane after they covered the entire plate. The genome was extracted using a NucleoBond HMW DNA kit (Macherey-Nagel, Düren, Germany). The *O. raphanipes* genome was sequenced using Illumina NovaSeq 6000 (paired-end, 2 × 150 bp) and Nanopore PromethION 48 sequencing platforms (Benagen Technology Co., Ltd., China, Wuhan), with a sequencing depth of ~60× for the Illumina platform and ~200× for the Nanopore platform. For RNA-Seq, total RNA was extracted from *O. raphanipes* mycelia and fruiting body using Trizol (Takara, Dalian, Liaoning Province, China) methods according to the manufacturer’s instructions. Paired-end sequencing libraries and analysis were performed on an Illumina NovaSeq 6000 platform (Illumina, CA, USA) by Benagen Co. (Wuhan, China).

### 2.3. Genome Assembling and Gene Prediction

The Nanopore reads were filtered using filtlong software and assembled using NECAT software [22]. The initial polishing was performed with racon v1.4.21 software (https://github.com/isovic/racon) for two rounds using Nanopore long reads. Then, pilon v1.24 was utilized to further correct the racon-corrected contigs with Illumina short reads for two rounds [23]. The assembly completeness was evaluated with QUAST v5.1.0rc1 software with the Illumina reads and Nanopore reads [24]. Gene prediction was performed by augustus software using *Laccaria bicolor* model [25] and by GeneMark-ES v 4.69 for self-training gene prediction. Meanwhile, the transcriptome data was filtered and assembled by trimmomatic, and Stringtie software was then re-trained by augustus for gene prediction. Finally, the software EVidenceModeler was used to integrate the gene prediction results mentioned above. Then the completeness of the assembled genome was also evaluated using BUSCO v5.1.2 software [26] with comparison to lineage dataset fungi_odb10 (creation date: 2020-09-10, number of BUSCO markers: 758). Protein functional annotation was performed using eggNGOmapper software [27], Pfam database, and SwissProt databases. In addition, gene clusters related to secondary metabolites were predicted by antiSMASH v6.0 [28]. Circular layouts were generated using Circos software (http://circos.ca/, accessed on 20 December 2022) [29]. 

### 2.4. Comparative Genomic Analysis

The pairwise average nucleotide identity (ANI) values between genomes were analyzed using FastANI software [30]. Collinearity analysis was performed using MCScanX software [31], based on location information from the GFF3 files of *O. raphanipes* CGG-A-s1, *Flammulina velutipes* (Genbank: PRJEB54953), and *Lentinula edodes* L808, as well as *O. raphanipes* CGG-A-s1, *Agaricus bisporus* var. *burnettii* H119, and *Pleurotus ostreatus* P15. Gene families and single-copy orthologous genes were analyzed using OrthoFinder v2.5.4 software as previously described [32]. Timetree was evaluated using single-copy orthologous protein sequences and performed using mcmctree software prom paml packages. The species tree was visualized using FastTree software. 

### 2.5. Repeat Sequence Identification

Repeat sequence was analyzed using RepeatModeler and RepeatMasker software [33,34]. RepBase database was used to predict sequences similar to known repeat sequences. RepeatModeler was used for de novo constructing the candidate database of repetitive elements, by which the repeated sequences were annotated using RepeatMasker (http://www.repeatmasker.org/RepeatModeler/, accessed on 22 December 2022).

### 2.6. Identification of CAZymes

Annotation of carbohydrate-active enzymes (CAZymes) for the genome of *O. raphanipes* was performed using dbcan version v3.0.2 software [35]. The database was downloaded from the dbCAN meta server (http://bcb.unl.edu/dbCAN2/, accessed on 28 December 2022) (version of the database is V10). Hmmer software was used for the annotation of proteins with default parameters (HMMER E-Value < 1e-15, HMMER Coverage > 0.35).

### 2.7. Analysis of Mating Genes

The mating genes from *Flammulina velutips* [36,37,38] and *Pleurotus giganteus* [15] were used as query sequences for the Blastp alignment against the proteome of *O. raphanipes* strain CGG-A-s1 and strain CGG-A-s2.

## 3. Results and Discussion

### 3.1. Genome Assembly of Monokaryotic O. raphanipes

**Figure 1:**
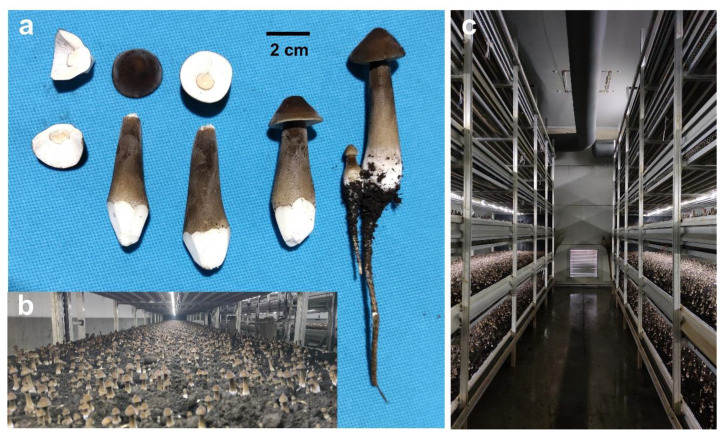
The fruiting bodies of *O. raphanipes* CGG-1. (**a**) The fruiting body morphology of *O. raphanipes*. (**b**) Industrial cultivation of *O. raphanipes*. (**c**) The greenhouse for industrial cultivation of *O. raphanipes*.

*O. raphanipes* is a well-known culinary edible mushroom that has been widely cultivated in China over the past few decades with the commercial name “Changgengu” or “Heipijizong”. We have succeeded in industrially cultivating the strain *O. raphanipes* CGG-1 in Shandong province (Figure 1). In this study, monokaryotic mycelia were germinated from the basidiospores of *O. raphanipes* CGG-1 by gradient dilution cultivation on the plate. Clamp connection was observed within the two monokaryons CGG-A-s1 and CGG-A-s2 after mating, which were used as sexually compatible monokaryotic strains and then for genome sequencing.

The genome of CGG-A-s1 was sequenced using Nanopore and Illumina sequencing platforms. A total 1,313,194 reads were obtained by Nanopore, with an average length of 9980 bp and a total length of 13.1 Gb, whereas 23,017,968 reads up to 3.44 Gb of data were obtained by Illumina using the PE150 method. Finally, 37 contigs with an N50 of 2.55 Mb and an N90 of 879 Kb were obtained after removing the repeats. The overall length was 60.2 Mb and the length of the largest contig was 5.27 Mb (Table 1). Repeat sequences accounted for 15.84% of the whole genome of CGG-A-s1 and the majority were LTR elements (5.60%), the LINE (0.04%), and simple repeats (0.30%) (Table 2). The integrity of the genome was evaluated using QUAST v5.1.0 software and determined to be 99.25%. In order to conduct comprehensive genomic prediction, the transcriptome sequencing was performed by Illumina using the PE150 method and a total of 95.5M reads up to 14.32 Gb of data were obtained. The whole genome of monokaryon CGG-A-s2 generated from the Illumina platform was assembled by SPAdes software and 3,154 contigs (>500 bp) were obtained. The total length was 54.9 Mb with an N50 of 77.4 Kb and an N90 of 9.94 Kb (Table 1). 

### 3.2. Gene Prediction of O. raphanipes

A total of 21,308 CDSs were predicted for the CGG-A-s1 genome with an average length of 1,406 bp. The cumulative length of encoded genes was 30.0 Mb, which accounted for 49.8% of the whole genome. The average exon and intron numbers per gene were 5.78 and 4.78, respectively (Table 3). The completeness of the *O. raphanipes* CGG-A-s1 genome assembly and gene prediction was evaluated by the BUSCO software, which was analyzed using the fungi_odb10 (758 genes) database with a completeness of 94.4%, the basidiomycota_odb10 (1764 genes) database with a completeness of 93.9%, and the agaricales_odb10 (3870 genes) database with a completeness of 91.3% (Figure S1). The results suggested that we presented a high-quality genome sequence of *O. raphanipes* with preferable integrity and continuity.

### 3.3. Genome Annotation of O. raphanipes

**Figure 2:**
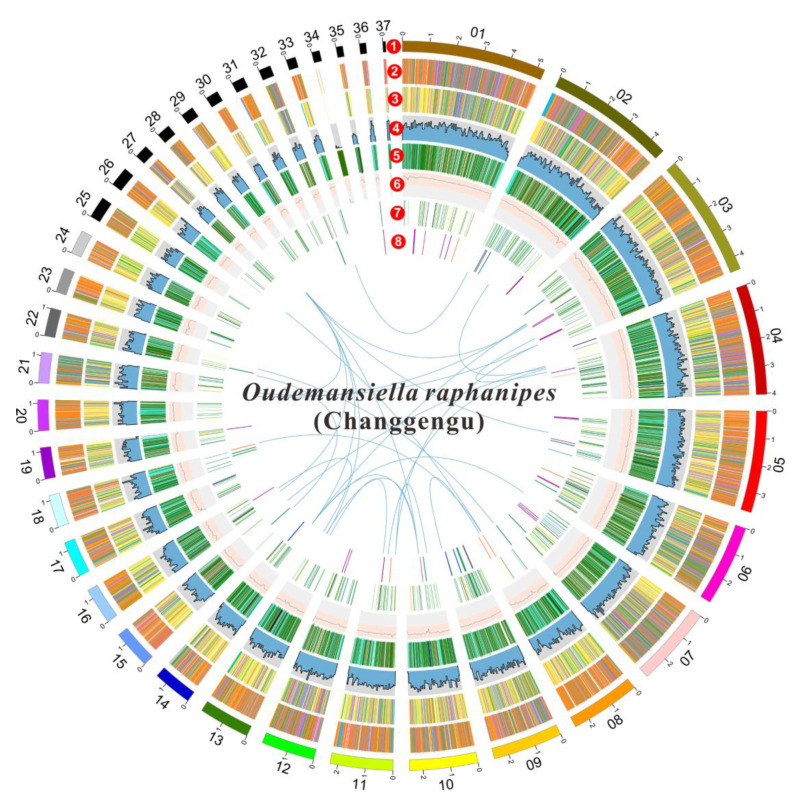
Circle map of the genome assembly and gene prediction of *O. raphanipes* CGG-A-s1. A total of seven layers were plotted from the outside to the inside. The outmost layer one is a circular representation of the 37 contigs with size intervals of 1 Mb. Layers two and three represent the predicted genes in the forward and reverse strands of the genome. Layer four indicates the gene density. Layer five indicates the repeat sequences. Layer six represents the GC content. Layer seven represents the genes of CAZymes. Links within and between chromosomes indicate collinear blocks generated from MCScanX: terpene (purple), NRPS or NRPS-like (green), fungal-Ripp-like (blue), T1PKS (orange), NRPS-independent-siderophore (red), indole (light orange), both of fungal-RiPP-like and terpene (light blue).

The predicted gene sequence was functionally analyzed by EggNOGmapper, SwissProt, Pfam, and CAZymes databases as shown in Table S1. Among the annotated genes, 15,004 and 7753 genes were classified by the EggNOGmapper database and SwissProt database, respectively. A total of 11,289 genes were annotated by the Pfam database via Hmmer based on the similarity of protein domains, and 1152 secreted proteins, containing a signal peptide without transmembrane domains, were identified. In addition, a total of 56 gene clusters related to secondary metabolites were predicted by antiSMASH software. Among them, there are 24 gene regions that are associated with terpenes, 16 gene regions that are involved in non-ribosomal peptide synthase (NRPS) or NRPS-like, six gene regions that are related to type I polyketide synthase (T1PKS), two gene regions that are associated with indole, two regions that are associated with siderophore, and five gene region that are identified as fungal-RiPP-like. One region was predicted to be both fungal-RiPP-like and terpene. Table S2 and Figure 2 show the summary of predicted metabolite gene clusters. Finally, all genomic information of *O. raphanipes* CGG-A-s1 was circularly mapped by Circos software according to the results of genome assembly and functional annotation (Figure 2). 

### 3.4. Genome Evolutionary and Comparative Analysis

**Figure 3:**
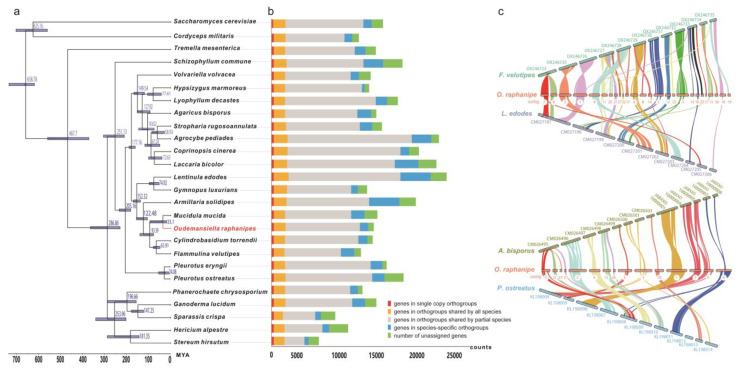
Evolutionary and comparative genomic analysis of *O. raphanipes*. (**a**) Phylogenetic tree was constructed based on 309 single-copy orthologs from *O. raphanipes* and 25 other fungal species (23 *Basidiomycetes* and 2 *Ascomycetes*) using OrthoFinder. Divergence timings were indicated using transparent blue bars at the internodes with 95% highest posterior density using MYA (million years ago) as a unit at the x-axis. Single-copy orthologs are defined as orthologs that were present as a single-copy gene in all 26 species. (**b**) Number of different orthologous gene types was calculated in each fungal species and indicated with different colors. (**c**) The genome collinearity among *O. raphanipes*, *F. velutipes* and *L. edodes* (upper), as well as *O. raphanipes*, *A. bisporus* and *P. ostreatus* (lower). Each line connects a pair of collinearity blocks between two genomes.

Phylogenetic analyses were carried out in order to investigate evolutionary relations between *O. raphanipes* and 25 other fungi species (23 Basidiomycetes and 2 Ascomycetes). 309 single-copy orthologous genes were found and used for phylogenetic reconstruction and species divergence time estimation (Figure 3). *O. raphanipes* was clustered in one clade with *M. mucid*, which indicated that they were closely related (Figure 3a). It was also shown that *O. raphanipes* had a close evolutionary relationship with cultivated edible mushrooms, such as *Flammulina velutipes*, Armillaria solidipes and Lentinula edodes. It was estimated that the divergence between species from *O. raphanipes* and *M. mucid* occurred ~33.1 million years ago (MYA). *O. raphanipes* split off from *F. velutipes* around 93.4 MYA, and it split off from *A. solidipes* and *L. edodes* around 122.5 and 152.3 MYA, respectively (Figure 3a). Considering that edible mushrooms with close relatives may be conserved in terms of cultivation methods and substrate utilization, the cultivation mode of *O. raphanipes* can be referred to as that of *F. velutipes* and *A. solidipes*.

For inter-species comparative genomic studies, the synteny of the *O. raphanipes* genome with two chromosome-scale assembled genomes in each of the two groups was analyzed. One group includes *F. velutipes* and *L. edodes*, the other includes *Agaricus bisporus* and *Pleurotus ostreatus*. Significant collinearity was detected between these species (Figure 3c). *O. raphanipes* and *F. velutipe* in the first group had the highest level of collinearity, covering 26 contigs, while *O. raphanipes* and A. bisporus in the second group had a smaller level, covering only 15 contigs. Based on the collinearity results from the two groups, the following contigs of *O. raphanipes* should belong to the same chromosome: contig 7 and contig 9; contig 11, 21, 27, and 32; contig 6, 8, and 14; as well as contig 15, 16, 22, and 31, in which 26 contigs of *O. raphanipes* CGG-A-s1 show collinearity with 12 chromosomes of *F. velutipes*. Rupture and fusion events were identified in contig 1, 2, 3, 5, 6, 7, and 9 of *O. raphanipes* compared to chromosomes from *F. velutipes* and A. bisporus.

### 3.5. ANI Value Analysis

**Figure 4:**
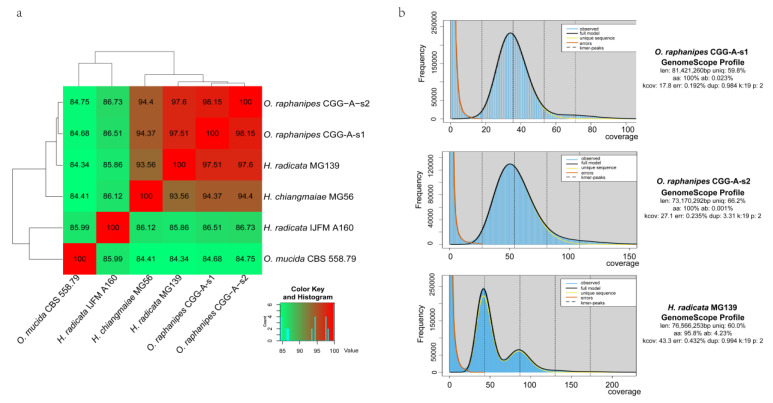
Genome assembly comparison within *O. raphanipes* and closely related strains. (**a**) Cluster heatmap of ANI values between six strains. (**b**) Histogram of the 19-mer depth distribution of the Illumina sequencing reads of *O. raphanipes* CGG-A-s1 and *H. radicata* MG139 plotted by GenomeScope. Blue areas indicate the observed k-mer frequencies and the black line indicates the fitted GenomeScope model.

Fungal identification is primarily based on phenotypic and physiological characteristics [39]. Changgengu has been confusedly classified as *H. raphanipes*, *H. furfuracea*, or *O. radicata* [2,5,9] due to a lack of morphologic and genetic information to verify the taxonomy. Many molecular methods have been developed for fungal identification, but the ITS (internal transcribed spacer) remains the key choice [39,40]. However, the ITS sequences cannot distinguish effectively at the species level. On the other hand, ANI values have commonly been used for species determination in prokaryotes on the genome scale [30]. Here, we adopted the ANI values to reflect the genetic correlation between the strains from the genus *Oudemansiella* and *Hymenopellis*. Three *Hymenopellis* strains and one *Oudemansiella* strain were extracted from the NCBI genome database in order to compare with the two monokaryotic *O. raphanipes* genomes assembled in this study. The ANI value between strain *O. raphanipes* CGG-A-s1 and CGG-A-s2 was 98.15%, which displayed the highest similarity. The ANI values between *O. raphanipes*, *H. radicata* MG139 and *H. chiangmaiae* MG56 are all greater than 93.56%, indicating a close taxonomy relationship within species. However, it is interesting to note that the ANI value between the two *H. radicata* strains themselves is only 85.8%, far below the threshold for intraspecies in prokaryotes, which is 95% (Figure 4a). The O. mucida CBS 558.79 strain displayed the lowest ANI values (~85%) with other species. It is worth noting that the ANI value between *O. raphanipes* CGG-A-s1 and *H. radicata* MG139 is 97.5%, suggesting that the two strains are probably from the same species. 

Although no benchmark analysis has been performed to evaluate the use of fastANI value in the taxonomy of fungi, considering the different genome sizes between fungi and prokaryotes, our results suggested that all six strains should belong to the same genus (Figure 4a) and four strains (CGG-A-s1, CGG-A-s2, MG139, and MG56) should belong to the same species.

Under these circumstances, high-quality genome sequencing and assembly level providing comprehensive instructions are more than necessary. The genome assembly level of strain *O. raphanipes* CGG-A-s1 was much better than that of strain *H. radicata* MG139, which was assembled into 31,379 contigs with an N50 of 6.07 Kb and an N90 of 0.92 kb [14]. In a word, further research should be performed to evaluate the digital DNA hybridization analysis methods in the taxonomy research of fungi.

Furthermore, Illumina reads were used for k-mer analysis using GenomeScope to generate a histogram of the depth distribution of the sequencing (k=19) (Figure 4b). A single k-mer coverage peak was observed for *O. raphanipes* CGG-A-s1 and CGG-A-s2. The heterozygous rate was 0.023% and 0.001%, respectively. Two major peaks between 42 and 85 were observed for *H. radicata* MG139, and the first peak is higher than the second with high heterozygosity (4.23%). This evidence demonstrates that strain *H. radicata* MG139 is a dikaryotic strain, while strain *O. raphanipes* CGG-A-s1 and CGG-A-s2 are monokaryons. However, with the rapid development of sequencing technology, more and more closely related species will be sequenced, which will make the taxonomic classification of Changgengu more accurate.

### 3.6. Carbohydrate Active Enzymes (CAZymes) in O. raphanipes Genome 

**Figure 5:**
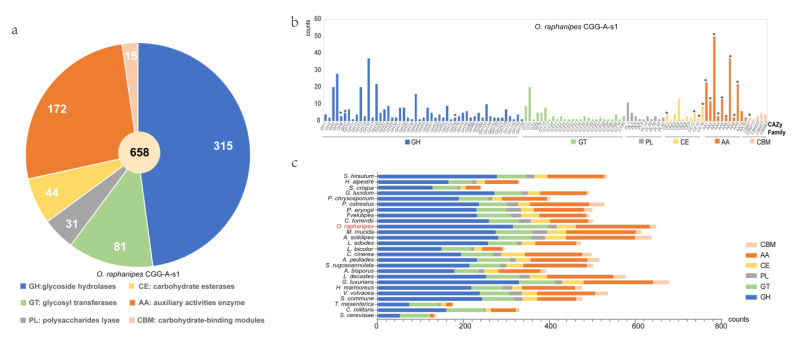
Distribution and number of carbohydrate-active enzyme (CAZymes) genes in *O. raphanipes* CGG-A-s1 and 25 other fungi. (**a**) Distribution of CAZymes in *O. raphanipes* CGG-A-s1. (**b**) Gene numbers of CAZyme families in *O. raphanipes* CGG-A-s1. The asterisks indicate the 17 gene families involved in the wood degradation. (**c**) Comparison of CAZymes in the 26 fungi. The strain names of these fungi are the same as those in Figure 3.

CAZyme analysis can provide insight into the metabolism of complex carbohydrates in *O. raphanipes*. In this study, the dbCAN2 database was used to identify 664 candidate CAZyme genes from the genome of *O. raphanipes* CGG-A-s1, including 320 glycoside hydrolases (GHs, 48.2%), 82 glycosyl transferases (GTs, 12.3%), 31 polysaccharides lyases (PLs, 4.7%), 44 carbohydrate esterases (CEs, 6.6%), 172 auxiliary activities enzymes (AAs, 25.9%), and 15 carbohydrate-binding modules (CBMs, 2.3%) (Figure 5a). Figure 5b shows the gene numbers of the corresponding CAZyme families. In terms of the genes related to wood degradation, 17 CAZyme families proposed by Floudas [12] were all found in *O. raphanipes* (Figure 5b). 

A comparison of the CAZyme profiles with 25 other fungi was also conducted, as shown in Figure 5c. The result showed that *O. raphanipes* CGG-A-s1 had the second highest number of CAZyme encoding genes, among which the GHs and AAs families were significantly elevated. Furthermore, the distributions of gene numbers in *O. raphanipes* CGG-A-s1 were also closely related to that of *M. mucida*. Genes belonging to the GH family have been observed to play an important role in the degradation of cellulose and hemicellulose, and even in the expansion of the cap and senescence of edible mushrooms [41,42]. The top three GH families (37 GH16 genes, 20 GH3 genes, and 22 GH18) genes identified were involved in hemicellulose digestion, which shows how efficient this process is. The AA-related genes in the *O. raphanipes* genome include AA1-AA9, AA14, and AA16 (Table S3, Figure 5b). Among them, the AA1 enzymes are multicopper oxidases that are involved in lignin degradation, and the class II lignin-modifying peroxidases (AA2) are also important for lignin degradation as markers to discriminate between white rot fungi and brown rot fungi [43]. A total of 23 AA1-related genes (3 AA1, 15 AA_1, and 5AA_2) and 12 AA2-related genes were found in the *O. raphanipes* genome, which indicates a prominent lignin degradation ability. In addition, 50 AA3 and 22 AA9-related genes were also identified in *O. raphanipes*, which are involved in cellulose and hemicellulose degradation [17]. The results are consistent with the strong ability of *O. raphanipes* to degrade wood.

### 3.7. Identification of the Mating Genes

**Figure 6:**
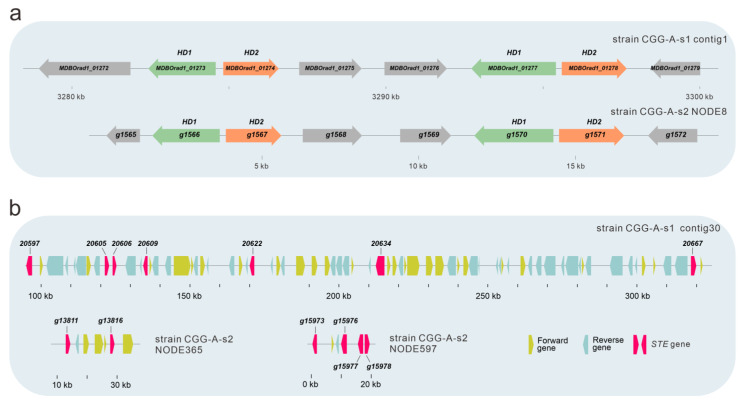
Gene structure of the mating type locus of in *O. raphanipes* CGG-A-s1 and CGG-A-s2. (**a**) Structure of A mating type locus in *O. raphanipes*. (**b**) Structure of B mating type locus in *O. raphanipes*.

Mating type recognition plays a crucial role in the genetics and breeding of mushrooms, influencing the propagating system, fruiting body, gamete quality, etc. [19]. The mating loci in two monokaryotic strains of *O. raphanipes* were identified by Blastp using mating genes from *F. velutips* [36,37,38] and *P. giganteus* [15] as query sequences. In the mating *A* locus of *O. raphanipes*, there are two clusters of homeodomain (HD) genes (Figure 6a). In one cluster, two *HD* genes (including one HD1 and one HD2 genes) were transcribed in opposite directions, as in many other mushrooms [15,37,44]. Both the mitochondrial intermediate peptidase (Mip) and the beta flanking gene (Bfg) were located in the two ends of the mating *A* locus of *O. raphanipes*. 

Sequence alignments showed that the mating *B* locus of *O. raphanipes* strain CGG-A-s1 was located in contig 30 (Figure 6b). Seven proteins with sequence similarity to the pheromone receptor (*STE*) genes from *F. velutips* [36,37,38] and *P. giganteus* [15] were identified in contig 30. Different from the mating *A* locus, the gene organization of the mating *B* locus in strain CGG-A-s2 was different from that in strain CGG-A-s1. Identified *STE* genes are located in different contigs. Two contigs containing more than one *STE* gene are shown in Figure 6b.

## 4. Discussion

Changgengu or Heipijizong is one of the most important commercial edible mushrooms in China, which has a long history in agricultural production, but a dispute over its scientific name. Though it has been assigned to the genus *Oudemansiella* according to the combined ITS and nrLSU [2], it was also classified as a species of other genera, mostly *Hymenopellis* [1,11]. Currently, a whole-genome similarity analysis has been one of the best ways to figure out the strain taxonomy and get genetic information on the molecular mechanisms of fungal growth and breeding [18,30,45]. However, few studies have investigated the genome sequence and genetic structure of *O. raphanipes*. Consequently, in this study, monokaryon genome sequencing was performed to explore the evolutionary status and genetic information of related functional genes of *O. raphanipes* CGG-1. With a combination of Nanopore and Illumina sequencing platforms, we assembled the genome of one of the monokaryons, *O. raphanipes* CGG-A-s1, into 37 contigs, which was much higher-quality sequencing data than its closely related reported strains, e.g., *H. radicata* MG139 and *H. radicata* IJFM A160. However, the ANI values indicated that our *O. raphanipes* strains and the other four strains should be interspecies or even intraspecies (ANI > 95) [30,46,47], which suggests that some of these strains are probably misnamed. Therefore, high-quality genome sequencing of more species within the genus is required in order to classify their taxonomic status. In order to comprehensively analyze the relationship between *O. raphanipes* and related species from other genera, 23 fungal species from Basidiomycota and 2 from Ascomycota were used for the phylogenetic analysis. *O. raphanipes* CGG-A-s1 was closely related to *M. mucid* (formerly also called *Oudemansiella* mucida), *Cylindrobasidium torrendii*, and *Flammulina velutipes* (one of the most widely cultivated mushroom species in China [48]). All these Agaricomycetes commonly have a small fruiting body, which are good resources for studying the underlying genomic changes in complexity level in mushroom fruiting bodies. Understanding the genetic bases of fruiting body evolution may directly contribute to the improvement of commercial mushroom production [49]. Furthermore, the synteny analysis showed high collinearity between the genomes of *O. raphanipes* CGG-A-s1 and *F. velutipes*, which suggests that *F. velutipe* probably served as a reference mode for cultivation and breeding of *O. raphanipes* during large-scale industrialization.

This study has identified many essential genes related to secondary metabolites, which endow *O. raphanipes* with biological activities that promote its survival in a given environment and defense responses to pathogens. Genes involved in the regulation of terpenes and NRPSs were mostly found in the genome of *O. raphanipes*. Terpenes are one of the largest groups of the bioactive natural products identified, which have been found in wild edible mushrooms and play an important role in biological functions [50,51,52]. For example, triterpenes from Ganoderma lucidum have been proven to have anti-cancer effects and can be used as a potential drug for cancer treatment [53]. It is worth noting that compared with other reported fungi, *O. raphanipes* is also predominate in the number of terpene synthesis genes [18,51]. NRPSs in fungi are considered to be corresponding to the virulence determinants during host-pathogen interactions, yet the exact role they play remains elusive in many cases [54,55]. To date, the function of NPRSs in Basidiomycetes has not been reported [19]. The expansion of terpenes and NRPSs in *O. raphanipes* suggests that it has significant potential for medicinal development.

CAZymes are important for the growth and development of wood-rotting mushrooms to thrive in environments rich in carbohydrates, especially lignocellulose and cellulose [18,56,57,58]. In the *O. raphanipes* genome, the GHs, GTs, and AAs were the main CAZymes, while PLs, CEs, and CMBs were in the minority. GH genes, the most highly elevated family in the genome of *O. raphanipes*, were four times more elevated than GT genes. This may be due to the lignocellulose degradation capacity that is necessary for the survival of *O. raphanipes*. Most GH-related genes are involved in starch degradation [41]. These evidences revealed that the enrichment of GH genes contributed to the diversification of nutrient substrate utilization for *O. raphanipes*. Moreover, compared with other fungi, *O. raphanipes* has the most AAs genes, which are important for lignin degradation [57,59].

*O. raphanipes* has the same gene organization structure as the mating A locus in other mushrooms [15,37,44]. Different mushrooms contain a different number of *HD* genes. *P. giganteus* and *P. ostreatus* PC9 only contain one pair of *HD* genes, while some contain three *HD* genes, such as those from *P. eryngii* [44], *Pleurotus diamor* [60], and *F. velutipes* [37]. Different monokaryotic strains from the same species may also contain a different number of *HD* genes. The strains of Hypsizygus marmoreus may contain three, four, or five *HD* genes [61]. As shown in Figure 6b, the two mating-compatible strains of *O. raphanipes* contain the same mating A structure with the same number of *HD* genes (Figure 6a). It is clear that the gene organization of *STE* genes on NODE365 and NODE597 are different from those in contig 30. Similar results have also been reported in other mushrooms, such as *F. velutipes* [15]. The gene organizations of *STE* genes in different mushrooms are quite different from one another [15,44,61]. All of the identified mating loci in *O. raphanipes* are based on sequence alignments. Nevertheless, the function of these genes in mating and clamp connection formation is still unknown. Further research should be conducted to determine the functions of these mating-related genes.

## 5. Conclusions

In summary, the whole-genome map of *O. raphanipes* was depicted for the first time in this study. Two monokaryons of *O. raphanipes* were isolated and used for sequencing by Nanopore and Illumina platforms. *O. raphanipes* CGG-A-s1 was sequenced with Nanopore and Illumina sequencing, generating 37 contigs with an N50 length of 2.55 Mb. *O. raphanipes* CGG-A-s2 was sequenced using the Illumina platform, obtaining 3154 contigs with an N50 of 77.4 Kb. The *O. raphanipes* CGG-A-s1 genome was functionally annotated using proprietary databases. The genome comparative analysis, genome evolutionary analysis, and CAZymes comparison expand our understanding of the taxonomic status, survival mechanisms, and capacity of *O. raphanipes*. Mating A and Mating B loci in both mating-compatible monokaryotic strains were identified, which provides useful information for the development of molecular markers for cross-breeding. The genome elucidation in this study provides foundational information and genetic resources for the exploration of metabolic compounds, industrial breeding, and genetic applications of *O. raphanipes*.

## Figures and Tables

**Table 1:** Genome assembly features of monokaryotic *O. raphanipes* CGG-A-s1 and CGG-A-s2.

Characteristics	Genome Aassembly Size (Mb)	Scaffolds	Contigs	Longest Scaffold (kb)	Scaffold N50 (kb)	Scaffold N90 (kb)	GC%	Sequencing Platform	Sequencing Date (Year)
*O. raphanipes* CGG-A-s1	60.2	37	37	5268.5	2552.6	879.0	50.2%	Nanopore, Illumina	2022
*O. raphanipes* CGG-A-s2	54.9	3154	3154	834.7	77.4	9.9	50.1%	Illumina	2022

**Table 2:** Repeat element analysis in the *P. giganteus* genome.

Repeat Elements	Copies (Numbers)	Repeat Size (bp)	Percentage of the Assembled Genome
LINEs/Bov-B	76	26,030	0.04%
LTR/Pao	131	51,290	0.09%
LTR/Copia	622	459,204	0.76%
LTR/Gypsy	1357	1,881,927	3.13%
LTR/others	2201	980,561	1.63%
DNA transposons/hobo-Activator	231	98,322	0.16%
DNA transposons/Tc1-IS630-Pogo	191	52,963	0.09%
DNA transposons/others	96	30,582	0.05%
Unclassified interspersed repeats	12,757	5,517,432	9.16%
Small TNA	26	1988	0.01%
Satellites	64	112,256	0.19%
Simple repeats	4380	181,298	0.30%
Low complexity	720	35,658	0.06%
Total	22,852	9,535,711	15.84%

**Table 3:** Characteristics of the gene prediction of *O. raphanipes* CGG-A-s1.

Content	Number/Length
Gene number	21,308
Average gene length (bp)	1406.18
Average protein length (aa)	467.73
Total exon number	101,884
Total exon length (bp)	29,962,853
Average exon length (bp)	294.1
Average exon number per gene	5.78
Total intron number	101,884
Total intron length (bp)	6,886,191
Average intron length (bp)	67.59
Average intron number per gene	4.78

## Data Availability

The whole genome sequence data reported in this paper have been deposited in the Genome Warehouse in National Genomics Data Center [62,63] under accession number GWHBRAH00000000 and GWHBRAI00000000, respectively, that is publicly accessible at https://ngdc.cncb.ac.cn/gwh, accessed on 1 December 22. The genome information of *H. radicata* MG139 and *H. chiangmaiae* MG56 can be acquired from NCBI database with Genbank Accession of GCA_003314005.1 and QLPD00000000, respectively. In addition, the data used to support the findings of this study are available from the corresponding author upon request (ftp://www.mushroomlab.cn, accessed on 1 December 22).

## Supplementary Materials

The following supporting information can be downloaded at: https://www.mdpi.com/article/10.3390/jof9020266/s1, Figure S1. BUSCO summary of *O. raphanipes* CGG monokaryotic strain CGG-A-s1. The completeness of CGG-A-s1 genome was evaluated using different databases as indicated on the y-axis. The blue area represents the number of complete (C) and single-copy genes (S), the dark blue area represents the number of complete and duplicated genes (D), and the yellow and red indicate the number of fragmented genes (F) and missing genes (M), respectively; and n indicates the number of all genes used; Table S1: Genome annotation of *O. raphanipes* CGG-A-s1 by EggNOGmapper, SwissProt, and Pfam databases; Table S2:Prediction of gene clusters for secondary metabolites in *O. raphanipes* CGG-A-s1; Table S3: Gene lists of CAZymes in *O. raphanipes* CGG-A-s1.
