# Supplementary material for: Whole Genome Sequence of an Edible Mushroom Oudemansiella raphanipes (Changgengu)
Source: J Fungi (Basel). 2023 Feb 16;9(2):266. doi: 10.3390/jof9020266 (PMC9961838; doi:10.3390/jof9020266)
Supplement: Supplementary file 1 [file jof-09-00266-s001.zip › Supplemental Materials-Figure S1.pdf]

Supplemental Materials

Whole Genome Sequence of an Edible Mushroom *Oudemansiella raphanipes* (Changgengu)

Liping Zhu[1,2 †], Xia Gao[3, †], Meihua Zhang[1], Chunhui Hu[1], Wujie Yang[3], Lizhong Guo[1], Song Yang[1], Hailong Yu[2, *] and Hao Yu[1,*]

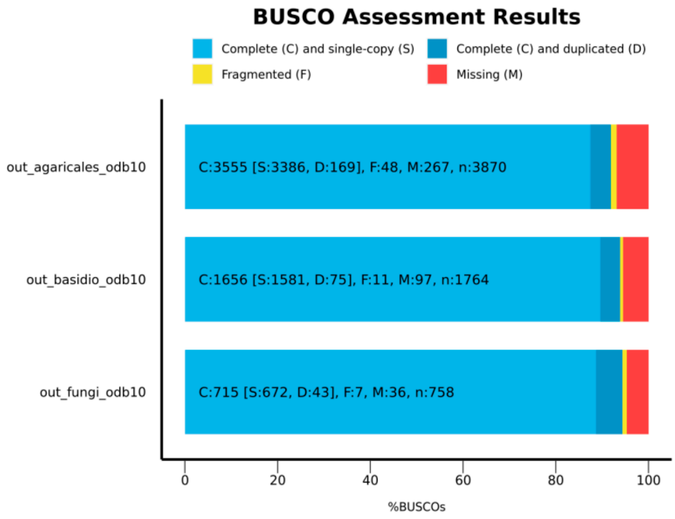

Figure S1. BUSCO summary of *O. raphanipes* CGG monokaryotic strain CGG-A-s1. The completeness of CGG-A-s1 genome was evaluated using different databases as indicated on the y-axis. The blue area represents the number of complete (C) and single-copy genes (S), the dark blue area represents the number of complete and duplicated genes (D), and the yellow and red indicate the number of fragmented genes (F) and missing genes (M), respectively; and n indicates the number of all genes used.
